# Investigating the impact of breastfeeding difficulties on maternal mental health

**DOI:** 10.1038/s41598-025-98357-6

**Published:** 2025-04-19

**Authors:** Gabrielle Rowles, Joseph Keenan, Nicky J. Wright, Karen Hughes, Rebecca Pearson, Hannah Fawcett, Elizabeth C. Braithwaite

**Affiliations:** 1 School of Psychology, Faculty of Health and Education, Manchester Metropolitan University, Manchester, UK; 2 School of Nursing and Public Health, Faculty of Health and Education, Manchester Metropolitan University, Manchester, UK

**Keywords:** Breastfeeding, Difficulties, Mental health, Postnatal, Behaviour change, Psychology, Human behaviour

## Abstract

Many women want to breastfeed but experience breastfeeding difficulties with associated consequences for poor mental health. The aim of the current study was to investigate mother’s perceptions of how breastfeeding difficulties have impacted their mental health, couched within a behaviour change model, to identify intervention targets. Data were collected in October 2023 via an online cross-sectional survey. Participants provided qualitative open-ended responses to describe how they felt that breastfeeding difficulties may have impacted their mental health (*N* = 1141). Data was analysed using thematic analysis and themes were mapped deductively on to the COM-B model of behaviour change. Mothers were highly motivated to breastfeed, but experienced challenges with psychological capability, physical capability, and social opportunity. Analyses resulted in the identification of the following targets for intervention: (1) education around the realities of what to expect when breastfeeding, (2) support for technical challenges and pain management, and (3) education for healthcare professionals, families and wider society on the importance of supporting breastfeeding mothers who are experiencing breastfeeding difficulties. Interventions targeted at breastfeeding mothers who are experiencing breastfeeding difficulties with associated mental health consequences have the potential to reduce rates of both early breastfeeding cessation and poor maternal mental health in the postnatal period.

## Introduction

The benefits of breastfeeding for both mothers and children have been widely documented^1–3^, and the World Health Organisation (WHO) and UNICEF (2003) recommend exclusive breastfeeding for six months and then continued breastfeeding with supplemental feeding for up to two years and beyond^2,4^. Despite this, breastfeeding rates in the UK are very low, whereby only 1% of mothers exclusively breastfeed to six months post-birth^4,5^.

There is a well-evidenced bi-directional link between maternal mental health and poor breastfeeding outcomes. Mothers with symptoms of depression or anxiety are less likely to initiate breastfeeding, have a shorter breastfeeding duration^6,7^, and experience more breastfeeding difficulties^8^ compared to mothers who do not have symptoms of depression or anxiety. Additionally, breastfeeding difficulties such as mastitis^9^, latching problems^10^, and severe breastfeeding pain^10,11^ can increase the risk for postnatal depression. However, there is very limited research utilising qualitative methods to explore how women make sense of how breastfeeding difficulties have impacted their mental health. Of the literature that does exist, there are some common themes that have been described. Mothers have reported that the reality of breastfeeding did not match their expectations^12,13^ and antenatal preparation set unrealistic expectations of breastfeeding^12^. Mothers felt uninformed by professionals about the difficulties that could occur when breastfeeding^14^ and expressed that professional discourse surrounding breastfeeding made them feel pressured to breastfeed^13^. Pain as a result of breastfeeding complications (e.g. mastitis, cracked nipples) was also reported in several studies to impact maternal mental health, as well as being a common reason for breastfeeding cessation^13–16^.

Helping mothers to manage the challenge of breastfeeding difficulties, therefore, has the potential to both reduce the early cessation of breastfeeding and improve postnatal mental health. Understanding how mothers perceive that breastfeeding difficulties impact their own mental health is an important first step toward reducing the impact of breastfeeding difficulties on maternal mental health. The aim of the current study is to advance current knowledge by mapping qualitative findings onto a model of behaviour change to aid in the identification of appropriate targets for intervention to reduce the impact of breastfeeding difficulties on maternal mental health. The COM-B (capability, opportunity, motivation – behaviour) model of behaviour suggests that a particular behaviour (in this case the continuation breastfeeding when experiencing breastfeeding difficulties and potential mental health consequences) will occur only when an individual has the capability and opportunity to engage in the behaviour, and the motivation to carry the behaviour out^17^.

## Method

### Design

A qualitative research design was used to understand women’s perceptions of how breastfeeding challenges have impacted their mental health. This research takes on the epistemological position of Interpretivism, as the aim is to understand perceptions based on women’s experiences of breastfeeding difficulties and resultant impacts on mental health^18^. The current study utilises data from a cross-sectional survey^19^, which was analysed using Thematic analysis (TA) and deductively mapped onto the COM-B framework. The survey was designed to examine associations between breastfeeding challenges and maternal mental health, and examine mediators and moderators of this association^20^. The survey was also designed to collect qualitative data to understand parents’ experiences of breastfeeding and impacts on their mental health, which is presented in this paper^20^.

## Sample

A total of *N* = 2010 participants completed an online survey in October 2023^19^. The participant inclusion criteria for the study was: over the age of 18, had first child within the past 10 years and gave their baby breastmilk. Participants were not eligible to take part in this study if they had given birth to multiples (e.g. twins, triplets), or if they had given birth before 37 weeks of gestation. Participant ages ranged from 20 to 53, the majority of the sample identified as White British (89.4%), and were highly educated, with 67.4% educated to undergraduate or postgraduate degree level. Participant demographic characteristics are displayed in Table 1. 87.9% of the sample indicated that they intended to breastfeed while pregnant, but only 39.4% reported that they had any knowledge of how to breastfeed before their baby was born. Just 52.4% of the sample reported that they received any support with breastfeeding during the postnatal period. Ethical approval for this study was granted by Manchester Metropolitan University Faculty of Health and Education Research Ethics Committee (REF 58254), and all methodological procedures were carried out in accordance with the relevant guidelines and regulations. All participants provided informed consent before participation in the research.

**Table 1:** Demographic characteristics of participants and their child.

Variable	*N* = 2010	%	Mean	Standard deviation
Maternal age	1991	–	34.59	5.104
Maternal ethnicity	2010	–	–	–
Asian or Asian British	86	4.3	–	–
Black/African/Caribbean/Black British	61	3	–	–
Mixed/Multiple Ethnic Groups	53	2.6	–	–
White	1797	89.4	–	–
Other	13	0.6	–	–
Maternal education	2005	–	–	–
Diploma	91	4.5	–	–
BTEC	60	3	–	–
GCSE	150	7.5	–	–
A Level	348	17.4	–	–
Undergraduate Degree	868	43.2	–	–
Postgraduate Degree	488	24.2	–	–
Childs age in months	2001	–	61.70	30.97
Childs age in years	2010	–	5.14	2.581

## Materials

Participants responded to the question ‘Do you think that breastfeeding challenges impacted your mental health? If yes, please describe’. *N* = 1172 (60.4%) participants responded yes, and *n* = 1141 (56.8%) participants provided a text response to describe how they felt that breastfeeding challenges impacted their mental health. These responses provide the qualitative data for the current study. All of the data from this study, as well as a data dictionary, script for data cleaning and imputation, and supporting study documents are openly available^19^.

### Data analysis

The data analysis followed the steps set out by Braun and Clarke^21^. The analysis began with familiarisation with the data by reading and re-reading the text data responses. Notes were taken throughout to identify initial patterns. Phase two involved generating initial codes; succinct labels for a feature of the data that was relevant to the research question were made^21^. Coding was completed once as many codes were identified to capture the diversity and the patterns within the data. Next, initial themes were generated based on the patterns identified from the coding process. The codes were deductively mapped onto the COM-B model of behaviour to identify themes relating to participant’s understanding of how breastfeeding challenges impacted their mental health, intention and ability to breastfeed. The COM-B model is composed of six components: reflexive motivation, automatic motivation, psychological capability, physical capability, social opportunity and physical opportunity^22^. As we undertook a deductive mapping approach using an existing model, there was limited data to support the presence of two of the COM-B components: automatic motivation and physical opportunity. Therefore, these themes are not present in the results section. Two authors (G.R. and E.B.) read and reviewed the quotes, discussed the themes, and any disagreements were resolved in consensus meetings.

To ensure methodological rigour, credibility and dependability in the analytical process, a diary was kept and used throughout all stages of the data analysis to bring awareness to any potential influences on the interpretation of the data. Additionally, an audit trail through detailed documentation of the data, research method and decisions made throughout analysis and writing process was kept, ensuring analytical dependability^23^.

## Results

Four categories within the COM-B framework were identified within the data: reflexive motivation, psychological capability. physical capability, and social opportunity. These categories are shown in Table 2 with indicative quotes.

**Table 2:** Summary of themes mapped on to COM-B framework.

COM-B Domain	Core themes	Data samples
Motivation	Maternal identity, motivation to breastfeed	I wanted to be able to breastfeed but baby wouldn’t latch, it made me feel as though I was doing it wrong but tried everything, I still think I should have maybe tried harder and persevered. I was very very upset when I made the decision to stop as I wanted to do it for much longer & I battled with the will to carry on and my pain and discomfort. Felt very guilty that I was unable to get baby to latch and feed as I really wanted to breastfeed It put alot of stress on me as I wanted to continue but it hurt and wasn’t going well. I tried my best and was proud that I managed a few months (and managed to express and feed breast milk in bottles alongside) but really regretful that I wasnt able to directly breastfeed longer I felt a compulsion to continue breastfeeding as I felt it was best for my baby.
Psychological capability	Lack of information, lack of skills	Significant impact on my mental health. I had no idea what I was doing and I still regret not breastfeeding for longer. I put so much pressure on myself and was so disheartened to find it was so much more difficult than I imagined. I felt very low due to the fact that breastfeeding was not the experience I had expected. My body wasn’t doing what it needed to do what was natural for it to do. I felt disappointed in myself and what I expected from breastfeeding I felt very low due to the fact that breastfeeding was not the experience I had expected…I felt like I was doing something wrong and it should be a lot more natural than it ended up being.
Physical capability	Technical challenges, pain	It was hard knowing that my milk supply was low…it made me really stressed and scared that he won’t get the necessary amount of milk to feel well fed and develop healthily. I had to resort to formula feeding. My milk did not come in straight away and I had to substitute formula a lot. When my milk did come in I was underwhelmed with how much I could pump and give my son, which gave me a lot of anxiety on how much my son was actually drinking straight from the breast so I was trying to pump a lot more to bring in more milk but it just wasn’t enough, it made me feel really upset that I couldn’t do that for him. She fed very frequently and when I had severe pain on latching when she was 8 months she was finally diagnosed with a tongue tie, but it was too late to sort I insisted on feeding even though I was experiencing a lot of pain because my child had undiagnosed tongue tie and I had to fight for 5 weeks to get this addressed and fixed. I had bleeding sore nipples and feeding was excruciating. Breastfeeding was excruciating as my baby was tongue tied. Various medical professionals told me my baby was fine and the pain of breastfeeding was all in my head…My nipples were so damaged that chunks of skin were falling off, and they were bleeding, but the breastfeeding expert at the hospital said she couldn’t understand what my problem was and my baby was feeding fine. I cried whenever I fed her for the first few days as it was so painful, and I would dread the next feed
Social opportunity	Lack of support from health professionals, fear of judgement from others.	My mood was extremely low, I felt abandoned by medical professionals who then judged me for not instinctively knowing how to breastfeed, I felt I was failing and alone I don’t feel like there was enough support from health care professionals. I felt very alone in the experience and that nobody was helping so this made me very stressed and in the end quite depressed Got awful advice and lack of support and was then threatened with hospitalisation to infant feeding support by the community midwife I was extremely stressed, depressed, alone in my struggles, unheard by midwives who were no help I hated my breastfeeding journey, I felt extremely judged from before I gave birth about my choices. I wish I had the strength as a first time mum to tell people to go away and keep their opinions to themselves I was un-informed and unsupported, and then felt judged when I actually started him on formula.

### Reflexive motivation

Generally, participants reported that they felt motivated to breastfeed and believed that breastfeeding was the best option for their baby.

I felt like this was what I was supposed to do. I felt pride and comfort.

Made me happy that I felt I was doing the best for my child.

Mothers’ motivation to breastfeed often resulted in many women describing that they felt upset or guilty when they could not breastfeed for longer than intended, or experienced challenges with breastfeeding.

Really regretful that I wasn’t able to directly breastfeed longer.

I felt so sad that I couldn’t breastfeed my baby for longer.

Many participants felt that breastfeeding difficulties were a threat to their maternal identity. This can be linked with motivation to breastfeed, because when mothers who intended to breastfeed found it difficult to establish or maintain breastfeeding, they associated this with their abilities as a mother.

I felt like I couldn’t do what was best for my baby, like [a] failure.

I was a bad mum who couldn’t even feed my child like I was supposed to.

Participants in this study were highly motivated to breastfeed. However, the combination of motivation to breastfeed and breastfeeding difficulties was a source of distress, because mothers were unable to do what they felt was best for their child.

## Psychological capability: the knowledge and skills to breastfeed

Participants reported a lack of knowledge of how to breastfeed, the realities of breastfeeding, and that breastfeeding expectations did not match their actual experience. Resultantly, mothers reported that they did not feel prepared for breastfeeding challenges, which in some cases, caused significant distress.

Breastfeeding was the hardest thing I have ever done in my life, both emotional and physically. I was not prepared for how difficult it was.

It was very traumatic for me. I didn’t even know that breastfeeding could be hard.

Some participants had no prior knowledge of how to breastfeed or what breastfeeding would be like, which led to feelings of being overwhelmed when experiencing breastfeeding difficulties.

I felt like I had failed my baby; he was my firstborn and I didn’t know what I was doing.

I had no idea what I was doing.

Participants indicated that they believed breastfeeding should be ‘natural’, and therefore instinctive. A substantial number of participants reported feeling that there was something wrong with them when they experienced breastfeeding difficulties.

You think it should be most natural thing but its actually very hard so makes you question everything.

Before my first child I expected it to be easy/come naturally. I just absolutely assumed I would be able to do it.

Prior to birth, participants were unprepared for the realities of breastfeeding, and had no knowledge that breastfeeding can sometimes be challenging. This means that when they did experience breastfeeding challenges, it induced feelings of psychological distress, overwhelm, and that ‘something wrong with them’ because of pre-existing perceptions of breastfeeding as ‘natural’ and ‘easy’.

## Physical capability: physical ability to breastfeed

Participants reported multiple technical challenges which impacted their ability to establish and/or maintain breastfeeding. Many participants reported difficulties with infant latching (64.4%), with some stating that they could not get their baby to latch at all, which seemed to impact mental health.

Made me feel depressed and a failure because I couldn’t get my son to latch properly.

I felt like a failure and a let down not being able to breastfeed as my son wouldn’t latch on.

Another challenge that many participants reported was perceived low milk supply (32.5%). Some mothers who reported a perceived low milk supply supplemented their infant’s feeding with formula, which resulted in feelings of guilt. Mothers also reported anxiety surrounding whether their baby was getting enough milk.

I was extremely anxious that my baby wasn’t getting enough milk.

I had low milk supply and had to supplement with formula. This made me feel I was lacking as a mother.

Many participants reported pain when breastfeeding (57.1%), which impacted participant’s ability to breastfeed and mental health. Participants whose babies were diagnosed with a tongue-tie reported breastfeeding to be painful. Additionally, complications such as mastitis and cracked nipples were also reported to be painful for mothers. Breastfeeding pain was a large contributor to early breastfeeding cessation, and participants stated that pain made them dread feeding, which therefore impacted their mental health.

It was so painful at the start that I dreaded feeding and hated doing it.

The pain of having cracked nipples was horrendous, so I felt incredibly sad because I would dread feeding my baby.

### Social opportunity: social factors that influence ability to breastfeed

Lack of support from healthcare professionals was a widely reported issue, as many participants reported feeling unsupported by midwives and health visitors when they experienced difficulties with breastfeeding. Participants felt professional support was insufficient, and some reported no support at all.

I never received proper one to one help when I was having problems and was just quickly shown and left alone as if it’s meant to be so easy.

I felt there was no support. If I went out seeking advice or support, I was told by healthcare professionals (midwives, health visitors and GPs) to just “stop” breastfeeding.

Participants also reported feeling judged by others and wider society. Fear of judgement impacted mental health, especially when breastfeeding in public, and some participants reported that this contributed to early breastfeeding cessation. Participants expressed feeling judged for decisions they made about breastfeeding, including the decision to stop breastfeeding, using a bottle, and/or using formula.

Pressure from society to breastfeed is enormous, so when someone asked if I’m breastfeeding I felt stress[ed] to tell that I’m not anymore.

I think it has made me angry at society that it isn’t more understood and supported.

There were therefore social factors that influenced both maternal mental health and breastfeeding cessation, including inadequate support from healthcare professionals, and a fear or judgement from other parents and wider society about their decisions to stop breastfeeding or to supplement infant feeding with formula.

## Discussion

This novel study examined perceived impacts of breastfeeding difficulties on maternal mental health. We inductively analysed qualitative data from over 1000 participants, and then deductively mapped the themes onto the COM-B model of behaviour change, to identify whether interventions to support mothers who are experiencing breastfeeding difficulties that are impacting their mental health, should target capability, opportunity, or motivation to breastfeed. Following this behavioural diagnosis, intervention designers can then identify appropriate intervention functions, mechanisms of change, and select specific behaviour change techniques with the aim of encouraging the continuation of breastfeeding.

Participants in this study were motivated to breastfeed, and often related breastfeeding to being a ‘good mother’, underpinned by the belief that breastfeeding was the ‘best thing’ for their baby. Similar sentiments were reported by Burns, et al.^13^; participants believed that the breastfeeding meant that they were a good mother. Motivation to breastfeed may therefore be detrimental to maternal mental health in the context of breastfeeding difficulties, because it undermines maternal identity.

Psychological capability, physical capability and social opportunity were reported to impact women’s ability to establish and/or maintain breastfeeding, and also participant mental health. NICE guidelines indicate that mothers should be provided with information and advice about milk supply, pain when breastfeeding and breastfeeding complications^24^. Despite this guidance, many participants in the current study reported a lack of skills and knowledge of how to breastfeed, with numerous participants expressing that their expectations of breastfeeding did not match their reality. The theme of ‘expectation vs. reality’ has been identified across previous studies, where mothers reported that they were not prepared for the difficulties they would experience while breastfeeding^12,13,15^. Lack of preparedness for breastfeeding difficulties was also reported by participants in the current study, and many mothers described this as a source of distress. Improved education for expectant parents on the reality of breastfeeding may therefore psychologically prepare mothers for breastfeeding challenges, which may resultantly prevent early breastfeeding cessation and reduce the negative mental health consequences of breastfeeding difficulties. However, an important consideration is that education around the realities of breastfeeding may induce or inflate anxieties in new parents about breastfeeding. It would therefore be important to co-produce educational materials with breastfeeding parents and healthcare professionals, and evaluate the impact of such educational resources on both parental mental health and breastfeeding rates.

Physical capability relates to the technical challenges of breastfeeding, and existing literature has highlighted perceived insufficient milk supply is the most common reason for formula supplementation^25^. Many participants in the current study reported that a low milk supply made them feel anxious that their baby was not getting enough milk, which caused them to supplement with formula. Previous research has shown that mothers felt relief once they had switched to formula, as they knew their baby was being fed sufficiently^26^. While mothers in this study communicated that they switched to formula to reduce anxiety surrounding milk supply, they also expressed guilt that they were not feeding their baby with breastmilk. Similar to previous studies^14,15,27^, breast pain was commonly reported in the current study, with some mothers describing the pain as ‘excruciating’ which caused them to ‘dread’ breastfeeding. Technical breastfeeding challenges and pain can therefore impact maternal mental health and undermine maternal identity while breastfeeding. Thus, interventions that aid new mothers with troubleshooting of technical challenges and pain management may benefit both breastfeeding maintenance and maternal mental health.

Mothers also reported that a lack of support from health professionals and a fear of judgement from others affected their ability to cope with breastfeeding difficulties. Similarly, Fox, et al.^12^ also reported that mothers expressed a dissatisfaction with routine care, citing that the advice they were given was often contradictory and inadequate. Thus, education on the importance of appropriate support for breastfeeding women who are experiencing challenges that may be impacting their mental health, targeted at healthcare professionals, family members and wider society may assist mothers with coping with breastfeeding challenges.

This novel study has generated valuable insights into the impact of breastfeeding difficulties on maternal mental health; a topic for which there is currently little knowledge. While the COM-B model is typically used to identify barriers and facilitators to a certain behaviour, its use in this study has allowed the identification of several potential targets for intervention to support women who are experiencing breastfeeding challenges which are impacting their mental health. Interventions should focus on: (1) education around the realities of what to expect when breastfeeding, including breastfeeding difficulties, (2) support for technical challenges and pain management when experiencing breastfeeding difficulties, and (3) wider education for healthcare professionals, families and society on the importance of supporting breastfeeding mothers who are experiencing breastfeeding difficulties.

Results of the study should be considered in light of the limitations. Findings may not be generalisable as the majority of mothers in this sample were white and highly educated, and we know that age, ethnicity and education are strong predictors of breastfeeding^28–31^. Additionally, though we had data from a large sample of participants, qualitative data came from just one free-text response question of an online survey, and therefore responses may not have been contextually detailed. Participants retrospectively reported on their breastfeeding experiences, which may be subject to recall bias.

## Conclusion

Breastfeeding and maternal mental health are globally recognised health goals, but in the UK breastfeeding rates are low and rates of poor maternal mental health are high. One potential factor related to both early breastfeeding cessation and poor maternal mental health is breastfeeding difficulties. In this novel study, we examined for the first time how mothers perceive that breastfeeding difficulties have impacted their mental health couched within a behaviour change framework. This allowed the identification of three targets for intervention for breastfeeding mothers who are experiencing difficulties. Such interventions may have the potential to reduce rates of both early breastfeeding cessation and poor maternal mental health in the postnatal period. As well as clear benefits for mothers, the benefits to child development are potentially twofold: via increased exposure to breastmilk and reduced exposure to poor maternal mental health.

## Data Availability

The dataset and supporting documents are available on the Open Science Framework, https://osf.io/3hwrx/.
